# Prevalence of Appropriate Testing for Incident Anemia in the US Department of Veterans Affairs

**DOI:** 10.1001/jamanetworkopen.2020.34406

**Published:** 2021-01-26

**Authors:** Andrew J. Read, Akbar K. Waljee, Charity S. Chen, Robert Holleman, Kyle E. Kumbier, Sameer D. Saini

**Affiliations:** 1 Division of Gastroenterology, Michigan Medicine, University of Michigan, Ann Arbor; 2 Institute for Healthcare Policy and Innovation, University of Michigan, Ann Arbor; 3 VA Health Services Research and Development Service, Center for Clinical Management Research, Ann Arbor, Michigan

## Abstract

This cohort study assesses the prevalence of appropriate testing for incident anemia in a large cohort from a national integrated health care system.

## Introduction

Anemia is a common problem that affects more than 5% of US adults, but the evaluation of new-onset (“incident”) anemia is not well standardized.^1^ Because anemia occurs frequently, clinicians may underappreciate its importance: up to 10% of adults with incident iron deficiency anemia (IDA) may have gastrointestinal cancer.^2,3^ Incident anemia thus requires prompt evaluation to prevent diagnostic delays. Because current practices for evaluation of incident anemia are unknown, we sought to characterize incident anemia evaluation in a national integrated health care system.

## Methods

The US Department of Veterans Affairs (VA) is the largest national integrated health care system and has robust laboratory and administrative data. We defined a national retrospective cohort among patients who received regular VA care (≥2 primary care visits in 2 years) and had incident anemia, defined by: (1) 2 or more normal hemoglobin (Hb) levels between January 1, 2013, and December 31, 2014; and (2) followed by anemia on 2 laboratory studies within 6 months of each other. The World Health Organization criteria were used for Hb levels: less than 12 g/dL for women and less than 13 g/dL for men (to convert Hb to grams per liter, multiply by 10). Patients were excluded if they were hospitalized within the 90 days before the incident date, or had laboratory evidence of anemia in the 5 years before the baseline (last normal) Hb level.

The primary outcome was appropriate testing within 1 year. Testing consisted of iron studies (ferritin, iron saturation, or both) in patients with microcytic anemia (mean corpuscular volume, < 80 μm^3^ [to convert to femtoliters, multiply by 1]); vitamin B_12_ and folate studies in patients with macrocytic anemia (mean corpuscular volume, >100 μm^3^); iron studies (ferritin, iron saturation, or both); and vitamin B_12_ and folate studies in patients with normocytic anemia (mean corpuscular volume, 80-100 μm^3^). Anemia was classified as mild, moderate, or severe using World Health Organization criteria (mild, Hb ≥11 g/dL; moderate, Hb ≥8 but <11 g/dL; or severe, Hb <8 g/dL). Iron deficiency anemia was determined using established likelihood ratios of ferritin.^4,5^ For incident IDA among patients who did not undergo esophagogastroduodenoscopy or colonoscopy in the preceding 2 years, we determined the proportion of patients who underwent endoscopic evaluation within the next year.

The VA Ann Arbor Healthcare System institutional review board approved the study and waived informed consent because the study posed minimal risk to participants and was not feasible without a waiver. This study followed the Strengthening the Reporting of Observational Studies in Epidemiology (STROBE) reporting guideline. Race was reported using patient-reported classifications. A 2-sided *P* < .05 was considered statistically significant. We used χ^2^ and Fisher exact tests for analysis of the categorical variables. Statistical analysis was performed using SAS, version 9.4 (SAS Institute Inc).

## Results

The cohort consisted of 49 648 patients, of whom 47 499 (95.7%) were men. The mean (SD) age of all patients was 71.8 (11.3) years (range, 23-100 years) (Table 1). In the year after the diagnosis of incident anemia, appropriate laboratory testing was performed in 15 592 of the 49 648 patients (31.4%) (Table 2). Among these patients, 2676 of 4013 (66.7%) had appropriate laboratory evaluation for microcytic anemia, 11 533 of 42 593 (27.1%) for normocytic anemia, and 1383 of 3042 (45.5%) for macrocytic anemia. The initial anemia severity, or alternatively, the change from the prior baseline normal hemoglobin value, was significantly associated with appropriate testing. Those with mild anemia were less likely to undergo additional evaluation (odds ratio, 0.53; 95% CI, 0.50-0.56; *P* < .001). There was no statistically significant difference in appropriate laboratory testing by race or sex when controlling for anemia severity. For those with IDA (ferritin level <35 ng/mL [to convert to micrograms per liter, multiply by 1) without recent endoscopic assessment, 3447 of 5050 patients (68.3%) did not undergo esophagogastroduodenoscopy or colonoscopy within 1 year of the diagnosis of incident anemia. Even with a lower ferritin cutoff value for IDA (<15 ng/mL; with an established likelihood ratio, 51.9; 95% CI, 41.5-62.3), 1777 of 2822 patients (63.0%) did not undergo endoscopic evaluation.^5^ Patients aged 50 to 75 years were more likely to undergo endoscopic evaluation for incident IDA compared with those younger than 50 or older than age 75 years (odds ratio, 2.1; 95% CI, 1.9-2.4; *P* < .001).

**Table 1.** Baseline Characteristics of the Study Population at the Time of Diagnosis of Incident Anemia

Characteristic	No. (%)		
	Men (n = 47 499 [95.7%])	Women (n = 2149 [4.3%])	Total cohort (N = 49 648)
Age, y			
Mean	72.4	58.1	71.8
Median (IQR)	72 (66-80)	59 (48-67)	71 (66-79)
Range	23-100	27-99	23-100
Race			
American Indian or Alaska Native	328 (0.7)	26 (1.2)	354 (0.7)
Asian	190 (0.4)	17 (0.8)	207 (0.4)
Black or African American	7182 (15.1)	597 (27.8)	7779 (15.7)
Multiracial	350 (0.7)	24 (1.1)	374 (0.8)
Native Hawaiian or Pacific Islander	377 (0.8)	17 (0.8)	394 (0.8)
White	36 215 (76.2)	1364 (63.5)	37 579 (75.7)
Missing/unknown	2857 (6.0)	104 (4.8)	2961 (6.0.)
VA practice type			
VA medical center	21 648 (45.6)	1034 (48.1)	22 682 (45.7)
CBOC	25 851 (54.4)	1115 (51.9)	26 966 (54.3)
Testing in prior 2 y			
FOBT or FIT	9597 (20.2)	385 (17.9)	9982 (20.1)
Colonoscopy	6192 (13.0)	237 (11.0)	6429 (13.0)
Esophagogastroduodenoscopy	2971 (6.3)	149 (6.9)	3120 (6.3)
Primary care visits, No./y			
Mean	4.9	4.9	4.9
Median (IQR)	4 (2.5-6)	3.5 (2.0-6)	4 (2.5-6)
Comorbidities			
Mean Charlson comorbidity index	1.53	1.04	1.51
Incident anemia severity^a^			
Mild	42 137 (88.7)	1571 (73.1)	43 708 (88.0)
Moderate	5091 (10.7)	558 (26.0)	5649 (11.4)
Severe	271 (0.6)	20 (0.9)	291 (0.6)
Anemia category^b^			
Microcytic	3690 (7.8)	323 (15.0)	4013 (8.1)
Normocytic	40 839 (86.0)	1754 (81.6)	42 593 (85.8)
Macrocytic	2970 (6.3)	72 (3.4)	3042 (6.1)
Change from baseline Hb level, g/dL			
Decrease of <1	13 063 (27.5)	710 (33.0)	13 773 (27.7)
Decrease of 1-2	17 574 (37.0)	828 (38.5)	18 402 (37.1)
Decrease of 2-3	8879 (18.7)	361 (16.8)	9240 (18.6)
Decrease of >3	7983 (16.8)	250 (11.6)	8233 (16.6)

Abbreviations: CBOC, community-based outpatient clinic; FIT, fecal immunochemical test; FOBT, fecal occult blood test; Hb, hemoglobin; IQR, interquartile range; VA, US Department of Veterans Affairs.

SI conversion factors: To convert the hemoglobin level to grams per liter, multiply by 10; mean corpuscular volume to femtoliters, multiply by 1.

^a^ Incident anemia severity was defined as follows: mild, Hb level of 11 g/dL or higher; moderate, Hb level of 8 g/dL or higher but less than 11 g/dL; and severe, Hb level less than 8 g/dL.

^b^ Microcytic anemia was defined as a mean corpuscular volume less than 80 μm^3^; normocytic anemia, 80 to 100 μm^3^; and macrocytic anemia, greater than 100 μm^3^.

**Table 2.** Testing Completed Within 1 Year of Incident Anemia in the Overall Cohort by Anemia Subtype and WHO Classification of Anemia Severity

Characteristic	WHO classification of anemia severity, No. (%) or No./total No. (%)^a^			Overall
	Mild	Moderate	Severe	
**Overall anemia cohort**	(n = 43 708)	(n = 5649)	(n = 291)	(N = 49 648)
Appropriate testing^b^	12 950 (29.6)	2489 (44.1)	1531 (52.6)	15 592 (31.4)
Men	12 474/42 137 (29.6)	2241/5091 (44.0)	143/271 (52.8)	14 858/47 499 (31.3)
Women	476/1571 (30.3)	248/558 (44.4)	10/20 (50.0)	734/2149 (34.2)
Age, y				
<50	391/1374 (28.5)	113/262 (43.1)	6/14 (42.9)	510/1650 (30.9)
50-74	7566/26 130 (29.0)	1503/3456 (43.5)	104/190 (54.7)	9173/29 776 (30.8)
≥75	4993/16 204 (30.8)	873/1931 (45.2)	43/87 (49.4)	5909/18 222 (32.4)
Laboratory tests				
Vitamin B_12_	20 005 (45.8)	2922 (51.7)	153 (52.6)	23 080 (46.5)
Folate	15 873 (36.3)	2488 (44.0)	125 (43.0)	18 486 (37.2)
Ferritin	17 334 (39.7)	3144 (55.7)	183 (62.9)	20 661 (41.6)
Iron saturation	16 593 (38.0)	3082 (54.6)	186 (63.9)	19 861 (40.0)
**Microcytic anemia**^c^				
Group total, No.	2839	1047	127	4013
Appropriate testing	1743 (61.4)	826 (78.9)	107 (84.3)	2676 (66.7)
Ferritin	1525 (53.7)	737 (70.4)	93 (73.2)	2355 (58.7)
Iron saturation	1484 (52.3)	724 (69.2)	97 (76.4)	2305 (57.4)
**Normocytic anemia**^c^				
Group total, No.	38 272	4176	145	42 593
Appropriate testing	10 035 (26.2)	1463 (35.0)	35 (24.1)	11 533 (27.1)
Vitamin B_12_	17 210 (45.0)	2077 (49.7)	64 (44.1)	19 351 (45.4)
Folate	13 510 (35.3)	1756 (42.1)	45 (31.0)	15 311 (36.0)
Ferritin	14 759 (38.6)	2194 (52.5)	79 (54.5)	17 032 (40.0)
Iron saturation	14 092 (36.8)	2158 (51.7)	79 (54.5)	16 329 (38.3)
**Macrocytic anemia**^c^				
Group total	2597	426	19	3042
Appropriate testing	1172 (45.1)	200 (47.0)	11 (57.9)	1383 (45.5)
Vitamin B_12_	1424 (54.8)	234 (54.9)	11 (57.9)	1669 (54.9)
Folate	1273 (49.0)	215 (50.5)	11 (57.9)	1499 (49.3)
**Iron deficiency anemia**^d^				
Group total	3764	1201	85	5050
Colonoscopy	939 (25.0)	364 (30.3)	28 (33.0)	1331 (26.4)
EGD	803 (21.3)	358 (29.8)	41 (48.2)	1202 (23.8)
EGD and colonoscopy	635 (16.9)	271 (22.6)	24 (28.2)	930 (18.4)
FIT or FOBT (and GI endoscopy)	424 (11.3)	197 (16.4)	14 (16.5)	635 (12.6)
FIT or FOBT (no GI endoscopy)	752 (20.0)	216 (18.0)	15 (17.7)	983 (19.5)
No endoscopic testing	2657 (70.6)	750 (62.5)	40 (47.1)	3447 (68.3)
Age, y				
<50	157/209 (75.1)	54/79 (68.4)	1/3 (33.3)	212/291 (72.9)
50-74	1304/2051 (65.6)	371/667 (55.6)	23/55 (41.8)	1698/2773 (61.2)
≥75	1196/1504 (79.5)	325/455 (71.4)	16/27 (59.3)	1537/1986 (77.4)

Abbreviations: EGD, esophagogastroduodenoscopy; FIT, fecal immunochemical test; FOBT, fecal occult blood test; GI, gastrointestinal; Hb, hemoglobin; WHO, World Health Organization.

SI conversion factors: To convert Hb to grams per liter, multiply by 10; mean corpuscular volume to femtoliters, multiply by 1.

^a^ Anemia severity is defined in a footnote to Table 1.

^b^ Appropriate evaluation was defined as (1) iron studies (ferritin, iron saturation, or both) for microcytic anemia; (2) vitamin B_12_ and folate studies for macrocytic anemia; and (3) iron studies (ferritin, iron saturation, or both), and vitamin B_12_ and folate studies for normocytic anemia.

^c^ Anemia subtypes are defined in a footnote to Table 1.

^d^ Patients with iron deficiency anemia group were defined as those with ferritin levels less than 35 ng/mL (corresponding to a likelihood ratio ≥2.5)^5^ without prior endoscopic assessment (no EGD or colonoscopy) in the past 2 years.

## Discussion

In this national retrospective cohort study, undertesting for incident anemia was common, and most patients with newly diagnosed IDA did not undergo endoscopic evaluation. However, this finding was attenuated by anemia severity; those with mild anemia were less likely to undergo evaluation, a finding similar to those in previous studies and health care settings.^6^ Potential limitations of our study include the possibility that (1) additional data were available to the clinician or were not captured (eg, menorrhagia, anemia of chronic disease, or alcohol-related anemia), (2) testing was ordered but not completed or performed outside the VA, or (3) there were contraindications to endoscopy (all of which would bias the results toward undertesting). Nonetheless, we attempted to mitigate these factors by selecting a cohort that received regular care, excluding those with potential iatrogenic anemia (occurring ≤90 days of hospitalization) and confirmed anemia (based on results of 2 separate Hb tests). It is important to note that the VA has made substantial efforts to assess and address potential diagnostic delays, such as those identified in this analysis. Future studies should examine variation in testing at the clinician level and identify further opportunities for intervention.
